# Full annual monitoring of Subantarctic *Emiliania huxleyi* populations reveals highly calcified morphotypes in high-CO_2_ winter conditions

**DOI:** 10.1038/s41598-020-59375-8

**Published:** 2020-02-13

**Authors:** A. S. Rigual-Hernández, T. W. Trull, J. A. Flores, S. D. Nodder, R. Eriksen, D. M. Davies, G. M. Hallegraeff, F. J. Sierro, S. M. Patil, A. Cortina, A. M. Ballegeer, L. C. Northcote, F. Abrantes, M. M. Rufino

**Affiliations:** 1 Área de Paleontología, Departamento de Geología, Universidad de Salamanca, 37008 Salamanca, Spain; 2 Antarctic Climate and Ecosystems Cooperative Research Centre, University of Tasmania, Hobart, Tasmania 7001 Australia; 3 CSIRO Oceans and Atmosphere Flagship, Hobart, Tasmania 7001 Australia; 4 National Institute of Water and Atmospheric Research, Wellington, 6021 New Zealand; 5 Institute for Marine and Antarctic Studies, University of Tasmania, Private Bag 129, Hobart, Tasmania 7001 Australia; 6 National Centre for Polar and Ocean Research, Vasco-da-Gama, Goa 403804 India; 7 Department of Environmental Chemistry, IDAEA-CSIC, 08034 Barcelona, Spain; 8 Portuguese Institute for Sea and Atmosphere (IPMA), Divisão de Geologia Marinha (DivGM), Rua Alferedo Magalhães Ramalho 6, Lisboa, Portugal; 9 CCMAR, The Centre of Marine Sciences, Universidade do Algarve, Campus de Gambelas, 8005-139 Faro, Portugal; 10 MARE - Marine and Environmental Sciences Centre, Faculty of Sciences, University of Lisbon, Campo Grande, 1749-016 Lisboa, Portugal; 11 Departamento de Didáctica de las Matemáticas y de las Ciencias Experimentales, Universidad de Salamanca, 37008 Salamanca, Spain

**Keywords:** Carbon cycle, Marine biology

## Abstract

Ocean acidification is expected to have detrimental consequences for the most abundant calcifying phytoplankton species *Emiliania huxleyi*. However, this assumption is mainly based on laboratory manipulations that are unable to reproduce the complexity of natural ecosystems. Here, *E. huxleyi* coccolith assemblages collected over a year by an autonomous water sampler and sediment traps in the Subantarctic Zone were analysed. The combination of taxonomic and morphometric analyses together with *in situ* measurements of surface-water properties allowed us to monitor, with unprecedented detail, the seasonal cycle of *E. huxleyi* at two Subantarctic stations. *E. huxleyi* subantarctic assemblages were composed of a mixture of, at least, four different morphotypes. Heavier morphotypes exhibited their maximum relative abundances during winter, coinciding with peak annual TCO_2_ and nutrient concentrations, while lighter morphotypes dominated during summer, coinciding with lowest TCO_2_ and nutrients levels. The similar seasonality observed in both time-series suggests that it may be a circumpolar feature of the Subantarctic zone. Our results challenge the view that ocean acidification will necessarily lead to a replacement of heavily-calcified coccolithophores by lightly-calcified ones in subpolar ecosystems, and emphasize the need to consider the cumulative effect of multiple stressors on the probable succession of morphotypes.

## Introduction

The Southern Ocean acts as a major sink for greenhouse gases by absorbing about one-sixth of anthropogenic annual emissions of CO_2_. However, this ecosystem service comes with a cost: enhanced CO_2_ absorption by the surface ocean rapidly alters seawater carbonate speciation, resulting in a decrease of carbonate-ion concentrations and pH, a process commonly referred to as ocean acidification. If anthropogenic emissions of CO_2_ continue to rise on current trends (i.e. under the IPCC IS92a “business-as-usual” scenario) surface seawater pH is predicted to drop by 0.3–0.4 units by 2100^1,2^. This magnitude and rate of decrease in pH are unprecedented in the past hundreds of millennia^3^ and are expected to have a profound impact on the marine environment^1,4^. Alterations in other environmental parameters are expected to co-occur with ocean acidification, including warming of the surface ocean, shallowing of mixed layer depths, changes in light, oxygen and nutrient supply and the southward migration of ocean fronts and circulation changes (see^5^ for a review). These environmental drivers may interact synergistically or antagonistically^6,7^, with largely unpredictable net effects on Southern Ocean ecosystems that need to be quantified^8,9^.

Coccolithophores are the most abundant group of marine calcifying phytoplankton and important contributors to the pelagic production of both particulate organic and inorganic carbon (POC and PIC, respectively). Satellite reflectance observations suggest the presence of high concentrations of PIC in the Southern Ocean attributed to elevated, seasonal concentrations of coccolithophores^10,11^. However, it should be noted that the satellite algorithm overestimates PIC concentrations in some regions, particularly in the Antarctic waters^12^. The seasonal presence of high coccolithophore accumulations may have profound and complex implications in carbon cycling of the region. On the one hand, calcite precipitation lowers total alkalinity and raises seawater partial pressure of CO_2_, thereby reducing the uptake capacity of CO_2_ by the ocean^13^. On the other hand, the ballasting effect of the high density coccoliths facilitates the sinking of associated organic matter to the ocean interior, thereby decreasing surface ocean partial pressure of CO_2_^14–16^.

*Emiliania huxleyi* (Lohmann) Hay et Mohler is the most abundant coccolithophore species in the Southern Ocean, where it displays a pronounced latitudinal gradient with maximum abundances in the northern-most provinces–including, the Subantarctic Zone–, and a stark decrease in abundance south of the Polar Front (PF)^17–19^. *Emiliania huxleyi* exhibits a range of morphotypes in the Southern Ocean (predominantly type A, A overcalcified, B, B/C and C) each of which with distinct coccolith morphology^20^, biogeographical distributions^17,21–23^ and with differences in light-harvesting-pigment and gene compositions among some of them^24,25^. Laboratory and field studies indicate that *E. huxleyi* may be susceptible to changes in environmental conditions elicited by climate change, particularly to ocean acidification^26–29^ and variations in nutrient supply^30^. However, due to their different physiological adaptations, the response of each of these morphotypes to environmental change is expected to be non-uniform^28^ with likely consequences on coccolithophore community composition and abundance, ultimately affecting the carbon cycle.

The Subantarctic Zone (SAZ) accounts for more than half of the areal extent of the Southern Ocean and is a globally significant region of water mass formation and anthropogenic CO_2_ uptake^31,32^. Predictions strongly suggest the SAZ waters will experience important changes in their physical and chemical properties including warming, freshening, acidification, enhanced delivery of iron-rich dust and shallowing of the mixed-layer depths, among others^5^. All these factors make the SAZ one of the high priority regions for sustained monitoring to relate ongoing environmental change to biogeochemical and biological activity.

**Figure 1:**
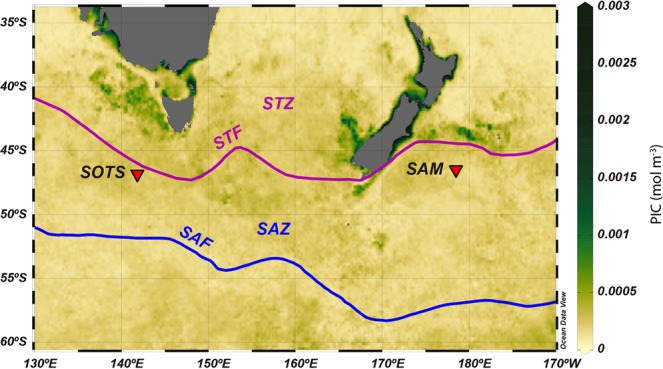
Location of the Southern Ocean Time Series (SOTS) observatory and Subantarctic Mooring (SAM) site (red triangles) superimposed on an annual composite (August 2011−August 2012) of MODIS PIC ocean concentrations. Abbreviations: STZ – Subtropical Zone, STF – Subtropical Front, SAZ – Subantarctic Zone, SAF – Subantarctic Front. Water masses and oceanic fronts after^35^. The software Ocean Data View (Schlitzer, R., Ocean Data View, odv.awi.de, 2018) was used to generate this figure.

The Southern Ocean Time Series (SOTS) observatory and the Subantarctic Mooring site (SAM) lie in the Subantarctic waters south west of Tasmania and south east New Zealand, respectively (Fig. 1). The SOTS and SAM sites have been instrumented quasi-continuously with deep-moored sediment traps (≥1000 m deep) since the late 90’s - early 2000s with the main objective of quantifying sinking carbon particle fluxes to the deep sea and for a broad range of biogeochemical studies^33,34^. Additionally, since 2010, the SOTS station has been equipped with a combination of moored platforms dedicated to meteorological and nutrient measurements and water sampling of the surface layer.

Here, we take advantage of the exceptional opportunity afforded by these monitoring tools to examine, with unprecedented detail, the seasonal variations of *E. huxleyi* populations in the Subantarctic Southern Ocean. We analyse water samples collected from the surface layer over a year (2011–2012) by an autonomous sampling platform in the surface layer of the SOTS site and explore their relationship with environmental parameters. Additionally, we examine the seasonal variations in abundance, composition and coccolith morphometrics of *E. huxleyi* sinking assemblages collected by three vertically-moored sediment traps deployed at SOTS during the same time interval and by a single sediment trap placed at the SAM site over a year (2009–2010). Our results reveal: (i) a consistent seasonal alternation of *E. huxleyi* morphotypes that is well correlated, although not necessarily causally related, with variations in the carbonate system and (ii) provide a quantitative and qualitative benchmark data of this keystone species against which future impacts of environmental change in the SAZ and wider Southern Ocean can be detected and measured.

## Material and Methods

### Sample collections and sensor measurements

The samples from Australia came from the SOTS observatory, which lies in the SAZ (near 47°S, 142°E), approximately 500 km south west of Tasmania^33^ (Fig. 1). SOTS was instrumented with three moored platforms: (i) a surface tower buoy that performs meteorological measurements (the Southern Ocean Flux Station - SOFS); (ii) a surface mixed layer mooring equipped with an automated water sampler) and nutrient, carbon and biological measurement sensors (the Pulse mooring); and (iii) a bottom-tethered deep sediment trap mooring that collects sinking particle fluxes for diverse biogeochemical studies (the SAZ mooring). The samples from New Zealand came from the deep-ocean SAM mooring deployed in Subantarctic waters south east of New Zealand (46°40′S, 178′ 30°E), and was equipped with sediment traps and a suite of sensors^36^ (Fig. 1).

Here, we document the *E. huxleyi* populations captured by sediment traps at ~1000, 2000 and 3800 m depth for a year from August 2011 until July 2012 at the SOTS observatory and a sediment trap at ~1500 m depth for a year from November 2009 until November 2010 at the SAM site. In addition, we examine *E. huxleyi* populations obtained with a McLane Remote Access Sampler (RAS) at ~34 m depth at the SOTS site. A detailed description of the remote sampler and sediment trap setup and physical and chemical analytical procedures together with information about the regional representativeness of the SOTS and SAM sites can be found in Supplement 1 and 2.

### Sample processing for coccolith analyses

All the sediment traps provided complete collection series, without any instrumental failures. Upon recovery, cups solutions were refrigerated, allowed to settle and aliquots of supernatant sampled with a syringe for salinity, nutrient and pH measurements to assess possible losses of their preservative-laden brines and thus the potential for sample degradation. This quality assessment step revealed no concerns. The remaining sample was wet-sieved over a 1 mm mesh sieve (SOTS) or a 200 µm sieve to extract zooplankton ‘swimmers’. Each sample slurry was then subsampled using a McLane wet sample divider; one tenth splits were made for the SOTS samples and one fifth splits for SAM.

A total of 80 sediment trap samples were prepared for coccolithophore analysis. The one tenth split dedicated to phytoplankton analysis from the SOTS sediment traps was further subdivided into four aliquots with the McLane splitter. One aliquot was used for calcareous nannoplankton analysis, other to measure pH before sample processing and the remaining two subsamples were kept refrigerated for future biomarker analyses. The pH of all samples was alkaline, ranging from 8.43 to 8.68, that is unfavourable to carbonate dissolution. For the SAM samples, a 1/25^th^ split was used for calcareous nannoplankton analysis.

For each trap sample, two glass slides were prepared using different methodologies. For the first type of preparation, a high volume (1000–5000 µl) of the raw sample was mounted on a glass slide following’s^37^ technique. This preparation was used for estimation of coccosphere fluxes and for coccolith morphometric analyses using the image processing software C-Calcita. Since algal aggregates, faecal pellets and coccospheres can contain large amounts of coccoliths, their presence in sediment trap samples can introduce substantial biases in the coccolithophore flux estimations^38^. In order to disaggregate the coccoliths contained in aggregates, and therefore obtain more accurate coccolith flux estimates, a modified protocol for non-destructive disintegration of aggregates from^38^ was followed for the preparation of the second glass slide. In short, 2000 µL were extracted from the split for nannoplankton analysis and were treated with 900 µL of solution of sodium carbonate and sodium hydrogen carbonate, 100 µL of ammonia (25%) and 2000 µl of hydrogen peroxide (25%) to remove organics. The sample was agitated repeatedly for 10 seconds every 10 minutes for a total period of one hour. Controls of the pH showed that the solution maintained a pH near 9, preventing coccolith dissolution. The reaction was stopped with catalase after an hour and samples were allowed to settle for at least 48 hours before preparation on glass slides.

A total of 24 sea-water samples collected by the RAS were processed for coccolithophore analysis. A volume ranging between ~180 and ~70 mL was filtered with polycarbonate filters (0.8 µm pore size; 13 mm diameter) using a low-pressure vacuum pump. After sample filtration, 200 mL of a buffer solution of sodium carbonate and sodium hydrogen carbonate (pH 8) was subsequently filtered in order to remove NaCl salts while optimizing coccolith preservation. The filters were then dried at ambient temperature and stored in Petri dishes. The filters were cut into two equal halves using a scalpel. One half-filter was mounted on an aluminium stub for *E. huxleyi* morphotype identification under the Scanning Electron Microscope (SEM). The coccoliths on other half filter were resuspended in a centrifuge tube through repeated washing and agitation on a vortex stirrer. The obtained slurry was then mounted on glass slides following the random settling method outlined by^37^.

### Microscopy

**Figure 2:**
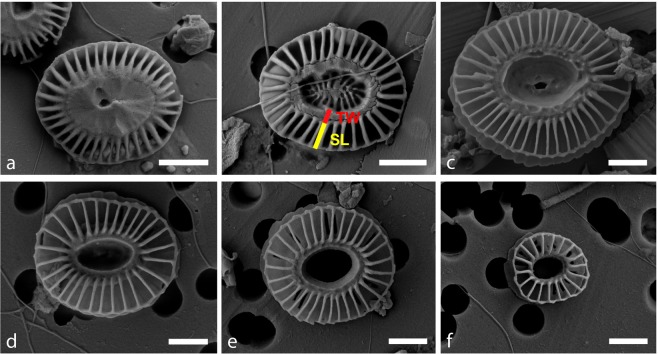
SEM images showcasing the five *Emiliania huxleyi* morphotypes found in the Subantarctic waters south of Tasmania and New Zealand: Type A overcalcified — A o/c — (**a**), A (**b**), B (**c**), B/C with central area covered by a thin plate (**d**), B/C with open central area (**e**) and C (**f**). SL = Slit Length (yellow bar), TW = Tube Width (red bar). Scale bars represent 1 μm.

In the sediment trap samples, coccospheres and coccoliths were identified to the lowest taxonomic level possible and counted using a Nikon Eclipse 80i polarised light microscope (LM) at 1000x magnification. All samples were also analysed under the SEM to identify *E. huxleyi* coccoliths to morphotype level. SEM samples were prepared using the same random settling method followed for the glass slide preparation. A Carl Zeiss EVO HD25 SEM was used to identify and classify a minimum of 100 *E. huxleyi* coccoliths into morphotypes found in the samples (magnification 5,000–20,000x). Main taxonomic features and representative images of each of the morphotypes identified in this study can be found in Table 1 and Fig. 2 and further details in Supplement 3.

**Table 1:** Classification of *Emiliania huxleyi* morphotypes in the Australian-New Zealand sector of the Subantarctic Zone (see Supplement 3 for further details).

Morphotype	Length of distal shield	Morphology of distal shield elements	Number of distal shield elements	SL/TW ratio	Morphology of central area and coccolith shape
Type A	2.5–4 μm	Moderate-robust calcified	26–36	>1	Clearly-visible central area elements
Type Ao/c	2.5–4 μm	Moderate-robust calcified	26–36	<1	Central area elements completely covered with thick plate or partially open
Type B	3.5–5 μm	Delicate/lightly calcified	≥35	NA	Open or sometimes covered with a thin plate
Type B/C	2.5–4 µm	Delicate/lightly calcified	25–33	NA	Open or sometimes covered with a thin plate
Type C	≤2–3.5 µm	Delicate/lightly calcified	18–25	NA	Open or sometimes covered with a thin plate. Coccolith shape often irregular compared to other morphotypes.

SL = slit length; TW = tube.

A target of 300 coccoliths and 100 coccospheres was established for the sediment trap sample analysis. Due to the strong seasonal fluctuations in coccolithophore production and export, there were some periods when almost no coccospheres were collected by the traps, and therefore the target of 100 coccosphere counted was not always accomplished. Coccolith and coccosphere counts were transformed into daily fluxes of specimens m^−2^ d^−1^ after^39^.

### Coccolith mass and size measurements

For determination of coccolith mass and length estimates of the sediment trap samples, the glass slide preparations of raw sediment trap material used for coccosphere identification were employed. A total of 2355 fields of view (FOV) were imaged using a Nikon Eclipse LV100 POL microscope equipped with circular polarisation and a Nikon DS-Fi1 8-bit colour digital camera. A detailed description of the circular polarization microscope set-up applied in this study can be found in^40^ while further details of the methodology used here can be found in Supplement 4.

### Statistics

Since the collection periods of the sediment traps were shorter than a calendar year, estimates of the annual *E. huxleyi* coccolith flux, coccolith mass and length and relative abundance of morphotypes were calculated to facilitate comparisons with other settings (Supplement 5).

Precise comparison of temporal changes in microplankton composition with the evolution of environmental drivers requires high-resolution sensor data coupled with sampling of the water column at discrete depths (e.g.^41^). Since such information is only available for the surface layer at the SOTS observatory (see Supplement 1 for details), the exploration of the relationship between environmental change and succession of *E. huxleyi* morphotypes was only conducted on this time series. While sediment trap records are a useful tool to monitor seasonal variations in microplankton composition two important factors introduce spatial and temporal uncertainties that hamper direct comparison of surface processes with particle fluxes registered by the traps. Firstly, the area of the surface ocean from which the coccolithophores assemblages have been produced increases with depth^42^. Therefore, although coccolithophores collected by the traps come from a region with similar seasonal cycle in water-column properties, it is likely that conditions were not identical, which would introduce some error in our analysis. Secondly, and most importantly, particle sinking rates in the Southern Ocean have been reported to exhibit pronounced seasonal variations (from 3 m d^−1^ in winter to up to 200 m d^−1^ in summer^43,44^). Although sediment trap records in our study region clearly mirror surface processes, the variable time lag of particle sinking rates throughout the year makes difficult to establish robust linkages between surface processes and particles fluxes measured by traps.

To explore the relationships between changes in the *E. huxleyi* morphotype relative abundance and environmental parameter variability measured in the surface layer at SOTS, two approaches were undertaken: a correlation plot with overlaid cluster analysis (non-directional) and a Canonical Correspondence Analysis (CCA) (constrained). For the correlation plot, variables were scaled prior to the analysis and spearman correlation coefficient was used, to account for monotonous relationships between variables (although the results were quite similar if the variables are not scaled or if Pearson coefficient was used instead). Correlations were considered significant when p < 0.05. For the canonical correlation analysis, morphotypes abundance were previously transformed using Hellinger transformation^45^. Akaike’s information criterion (AIC) was applied to identify the model with the minimum number of environmental variables that, being statistically significant explained the maximum inertia. Thus, a full model with all environmental variables (PAR, salinity, temperature, phosphate, TNOx – Total oxidised nitrogen –, silicate, TCO_2_, Ωcalcite, and pH) was first produced and subject to a stepwise variable selection procedure using AIC, before ordination analysis. Collinearity among predictors was tested using variance inflation factor analysis (VIF) and the model readjusted accordingly. Significance of the final model and the number of axes to be considered was estimated using permutation. All analysis were done using Vegan package^46^.

The relationship between coccolith mass and length at the two sites was modelled using a linear model. The full model (Mass = Length × Site) was subject to a model selection procedure. In order to explore which of the morphotypes was most representative of the mass, a multiple linear regression model (lm) was carried out. The best combination of explanatory variables was estimated by a step wise procedure, using AIC. All analysis and plots were produced using r-project.

## Results

### *Emiliania huxleyi* assemblages in the surface layer of SOTS

**Figure 3:**
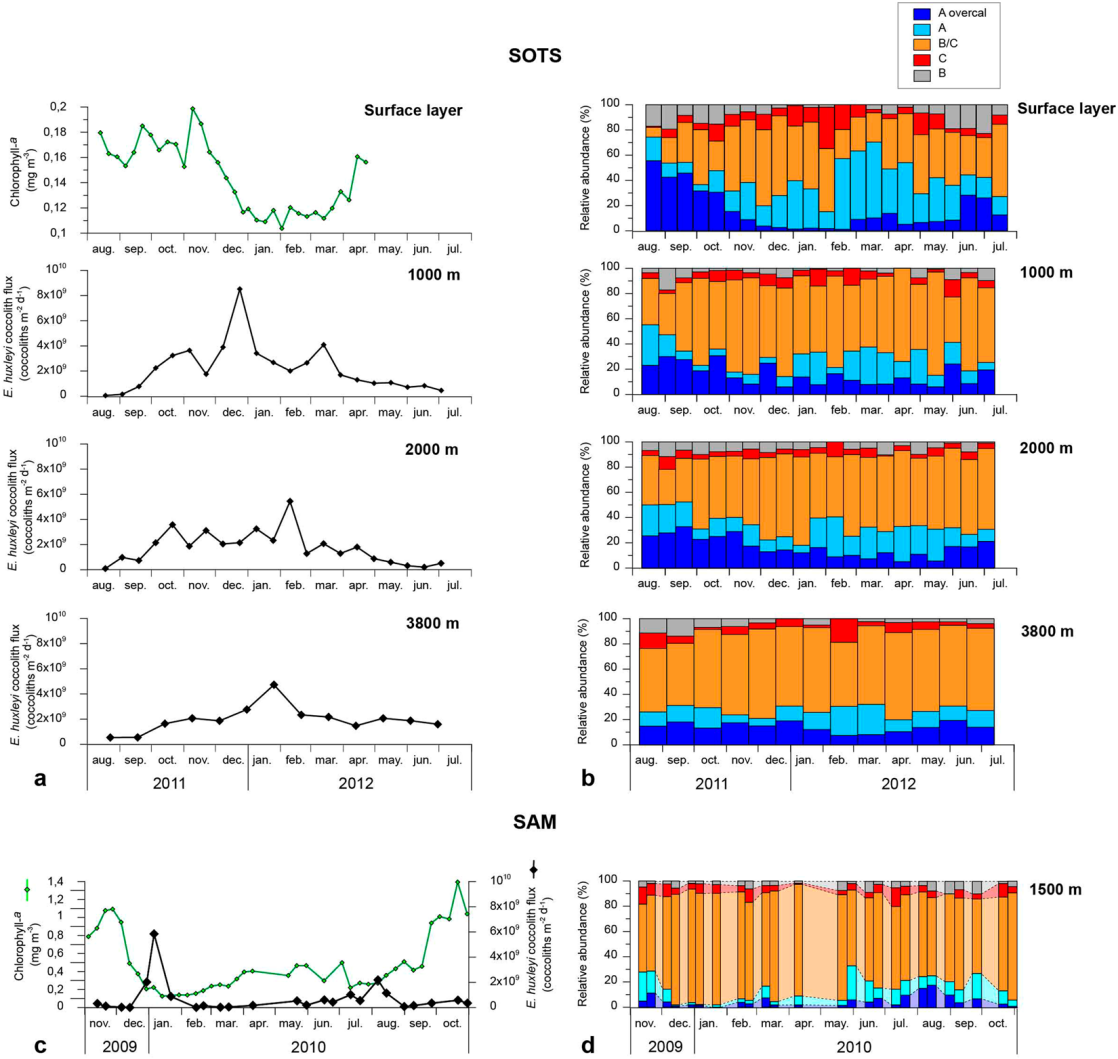
Seasonal variation on the satellite-derived chlorophyll-*a* concentration estimates, total *E. huxleyi* coccolith fluxes and relative abundance of *E. huxleyi* morphotypes at the SOTS (**a**,**b**) and SAM sites (**c**,**d**). Light coloured areas in (**c**) represent gaps in the time-series filled with linearly interpolated values.

Coccolith concentration in the RAS water samples was extremely low but sufficient to identify 100 *E. huxleyi* coccoliths in most of the samples. In only the first and last two samples of the record this target was not met, with 0, 92 and 96 coccoliths identified. The anomalous absence of coccoliths, other phytoplankton remains or detritus in the filter of the first sample was interpreted as an error during sample preparation or preservation issue and therefore, this sample was not considered in the analysis. Coccolith distribution on the half filters was largely uneven which precluded the estimation of absolute coccolith abundances in the water samples. Due to the very low number of coccoliths on the filters, the resuspension of the coccoliths trapped in the half filters dedicated to birefringence analysis was unsuccessful and therefore it was not possible to obtain coccolith mass estimates from the surface water samples. Therefore, only relative abundances of *E. huxleyi* morphotypes for the RAS samples are reported here (Fig. 3).

Detailed taxonomic analysis revealed a mixture of five *E. huxleyi* morphotypes in the surface waters south of Australia: Type A, A overcalcified (o/c hereafter), B, C and B/C. Type B/C was the most abundant morphotype (38.9 ± 14.2%; annual average ± standard deviation) for the sampling period, followed by A (26.9 ± 15.9%), Type A o/c (16.1 ± 15.8%), C (9.4 ± 7.2%) and B (8.7 ± 7.1%) (Fig. S1). The seasonal changes in morphotype abundance are described in section 3.4.

### *Emiliania huxleyi* sinking assemblages captured by the SOTS and SAM traps

*Emiliania huxleyi* coccolith fluxes collected by the sediment traps at the SOTS and SAM sites are represented in Fig. 3. Annualized *E. huxleyi* coccolith fluxes at the SOTS sediment traps (7.5, 6.0 and 6.9 × 10^11^ liths m^−2^ yr^−1^ at 1000, 2000 and 3800 m, respectively) were three-fold more than those estimated at the SAM trap (2.3 × 10^11^ liths m^−2^ yr^−1^). The contribution of intact *E. huxleyi* coccospheres to the total coccolith export was small, with annualized coccosphere flux about three orders of magnitude lower than that of coccoliths at the three depths of the SOTS site (1.6, 1.5 and 0.5 × 10^8^ coccospheres m^−2^ yr^−1^, respectively) and the SAM site (2.0 × 10^8^ coccospheres m^−2^ yr^−1^) (Fig. S2).

*Emiliania huxleyi* coccolith fluxes displayed a pronounced seasonality that was in line with the annual cycle of algal biomass accumulation in the surface layer, based on satellite remote-sensing data (Fig. 3b,d). At the SOTS 1000 m trap, coccolith fluxes started to increase in early October reaching maximum annual fluxes in late December 2011, and then decreased towards the winter. Coccolith fluxes at 2000 and 3800 m roughly followed the seasonality of the 1000 m trap (Fig. 3b). Seasonality in *E. huxleyi* coccolith flux at SAM was relatively similar to that of SOTS but with some differences: the period of enhanced coccolith fluxes was limited to December and January and a weak secondary maximum was registered in August (Fig. 3d). Total coccosphere fluxes at both sites also exhibited maximum fluxes during summer and minima during winter but peak coccosphere fluxes did not always coincide with maximum coccolith export (Fig. S2).

The annualized flux-weighted relative abundance for all the morphotypes indicate that *E. huxleyi* assemblages at the three sediment traps of SOTS were dominated by Type B/C (55–63%; relative abundance range for the three depths), followed by A (13–18%), A o/c (13–15%), C (6–7%), and B (4–6%) (Fig. S1). For the SAM site, the annualized flux-weighted *E. huxleyi* assemblages were also dominated by type B/C (79%), followed by C (7%), A (6%), A o/c and B (both 4%).

The temporal variations in the relative abundance of morphotypes registered by the SOTS sediment traps followed a general similar seasonal trend as that observed in the surface layer (Fig. 3a). At the SAM site, type B/C dominated the assemblages throughout the year reaching maximum relative abundances (>70%) in summer and autumn (i.e. December 2009 through April 2010) (Fig. 3d). Type A reached its maximum relative abundance in autumn (up to 27% in May-June). A o/c and B morphotypes displayed their highest relative contribution during the winter-spring transition (up to 18% in August 2010 for A o/c and 10% in August-September 2010 for B) and lowest during summer and autumn (down to 0% in January 2010 for A o/c and <2% in December 2009 and April and May 2010 for B) (Fig. 3d).

### Coccolith morphometric analysis

**Figure 4:**
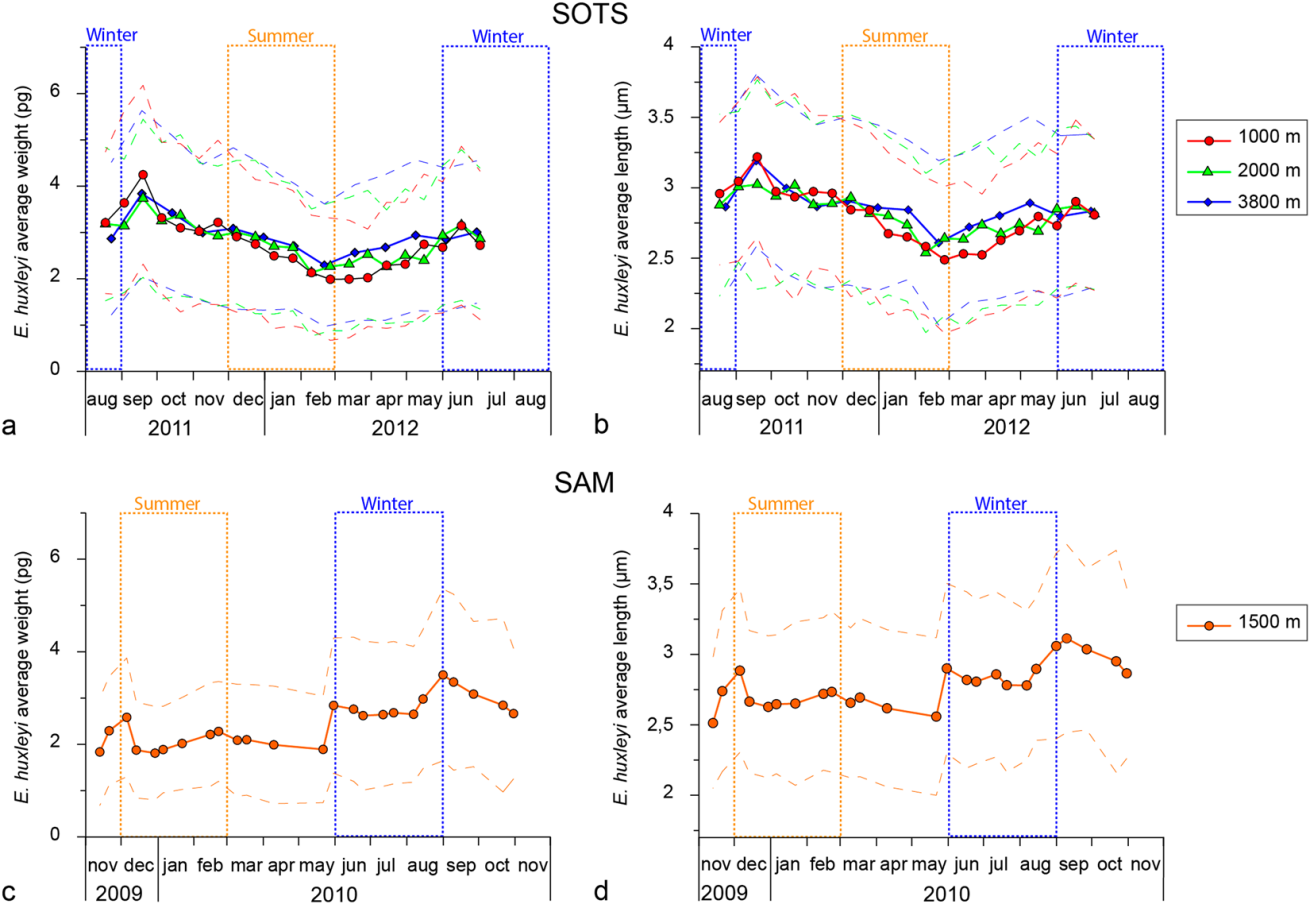
Average coccolith weight (**a**) and length (**b**) measured on the SOTS sediment trap samples collected between August 2011 to July 2012 at 1000, 2000 and 3800 m depth. Average coccolith weight (**c**) and length (**d**) for the SAM sediment trap (1500 m depth) between November 2009 to November 2010. Solid lines represent the average value for each sample while the dashed lines represent one standard deviation on each side of mean. Dashed boxes represent the austral winter (June–August) and summer (Dec–Feb).

While a target of 100 *E. huxleyi* coccoliths per sample was established, often many more were counted with an average of ~180 specimens per sample and a total of 14,241 for the four sediment trap records. The only exception were four samples from the SAM time-series where between 81–96 coccoliths were measured. The annualized flux-weighted average of *E. huxleyi* coccolith mass was similar between at all the SOTS traps with 2.67 ± 1.49, 2.75 ± 1.49 and 2.85 ± 1.52 pg (average ± standard deviation) at 1000, 2000 and 3800 m, respectively, compared to 2.28 ± 1.24 pg at 1500 m depth at SAM. The annualized weighted average of *E. huxleyi* coccolith length was 2.77 ± 0.56, 2.79 ± 0.59 and 2.84 ± 0.57 µm, respectively, compared to 2.73 ± 0.55 at SAM. Average coccolith mass of *E. huxleyi* populations displayed a pronounced and consistent seasonal cycle at meso- and bathypelagic depths of Subantarctic waters south of Australia and New Zealand (Fig. 4 and Supplement 6). Peak coccolith mass and length were observed during the winter/spring transition and were lowest during late-summer/early-autumn in all the sediment trap records from both sites (Fig. 4).

### Correlation and canonical correspondence analyses

**Figure 5:**
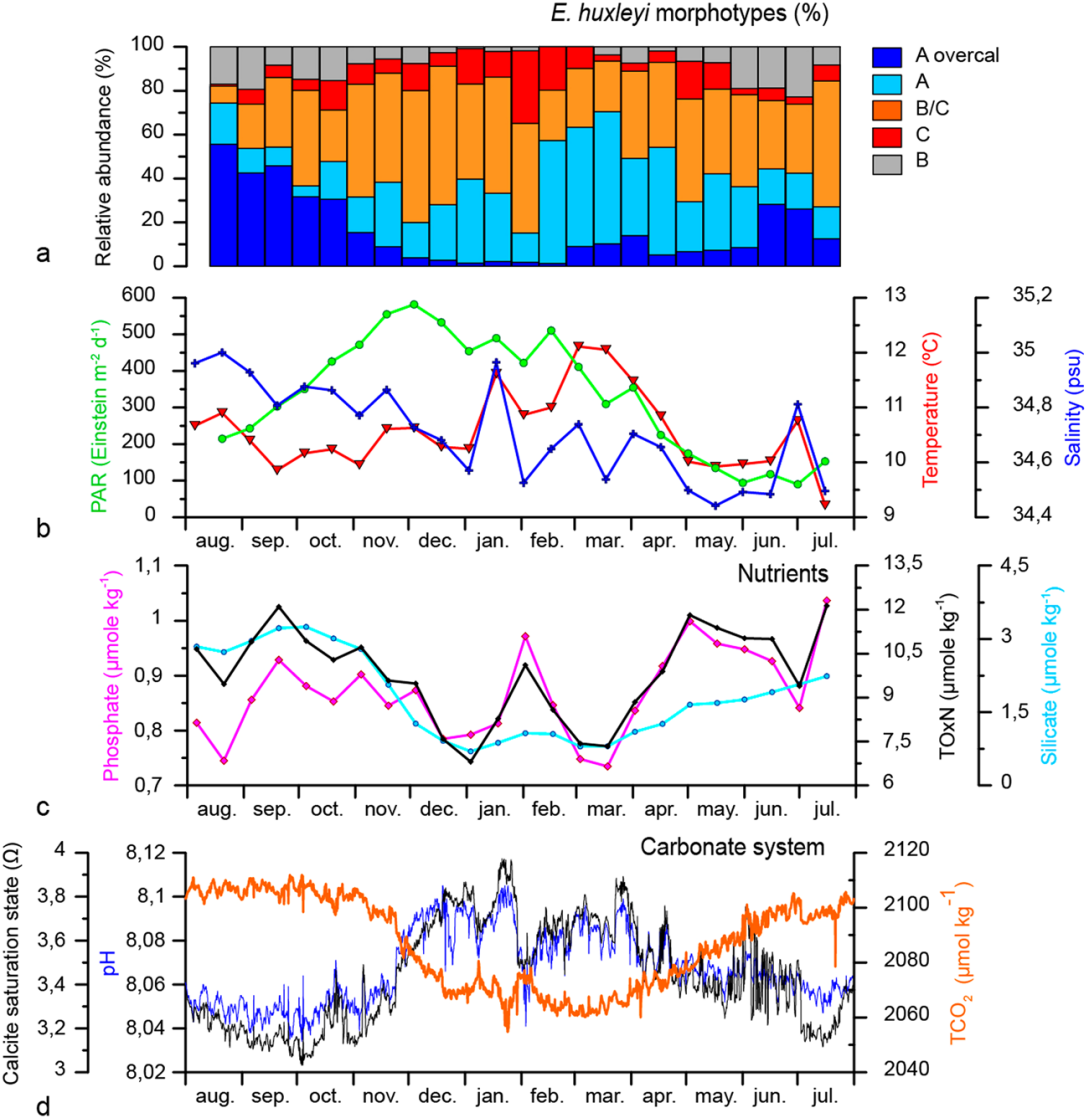
Relative abundance of the five *E. huxleyi* morphotypes (A overcalcified, A, B/C, C and B) collected by the autonomous water sampler (surface layer) at the SOTS site (**a**). Mean sea water temperature (45 m depth), Photosynthetically available radiation (PAR at surface), salinity (34 m depth) (**b**). Phosphate, Total oxidised nitrogen (TOxN: nitrate plus nitrite), and silicate concentration (34 m depth) (**c**). Carbonate system parameters after^47^ (**d**).

**Figure 6:**
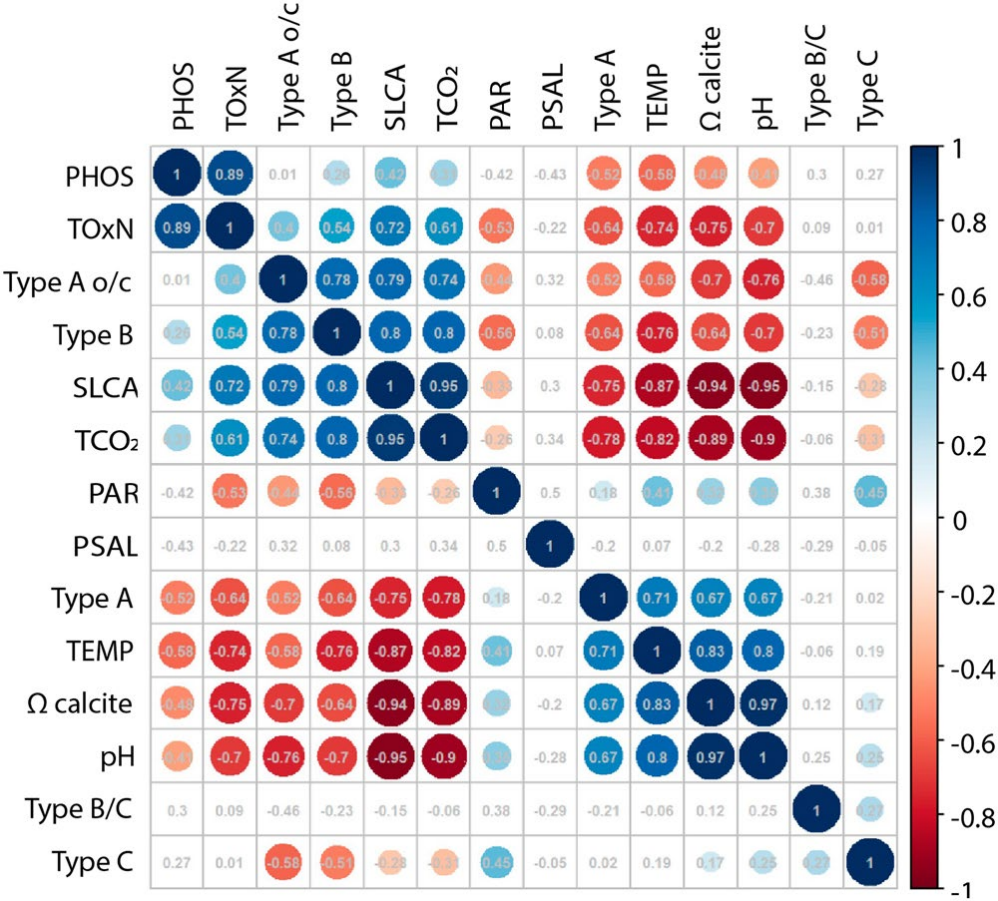
Spearman rank correlation matrix for environmental parameters and morphotype relative abundance in the surface layer at the SOTS observatory.

Overall, some of the environmental variables were strongly correlated between each other, in particular TCO_2_, pH, Ωcalcite and silicate concentration and also TNOx and phosphate (Figs. 5 and 6). The correlation matrix plot showed that Type A was positively correlated with temperature, Ωcalcite and pH. Type B and Type A o/c were mostly associated with high silicate concentration and TCO_2_, whereas type B/C and type C were not clearly associated with any of the measured environmental variables. PAR and salinity were also not correlated with any of the other variables (Fig. 6).

**Figure 7:**
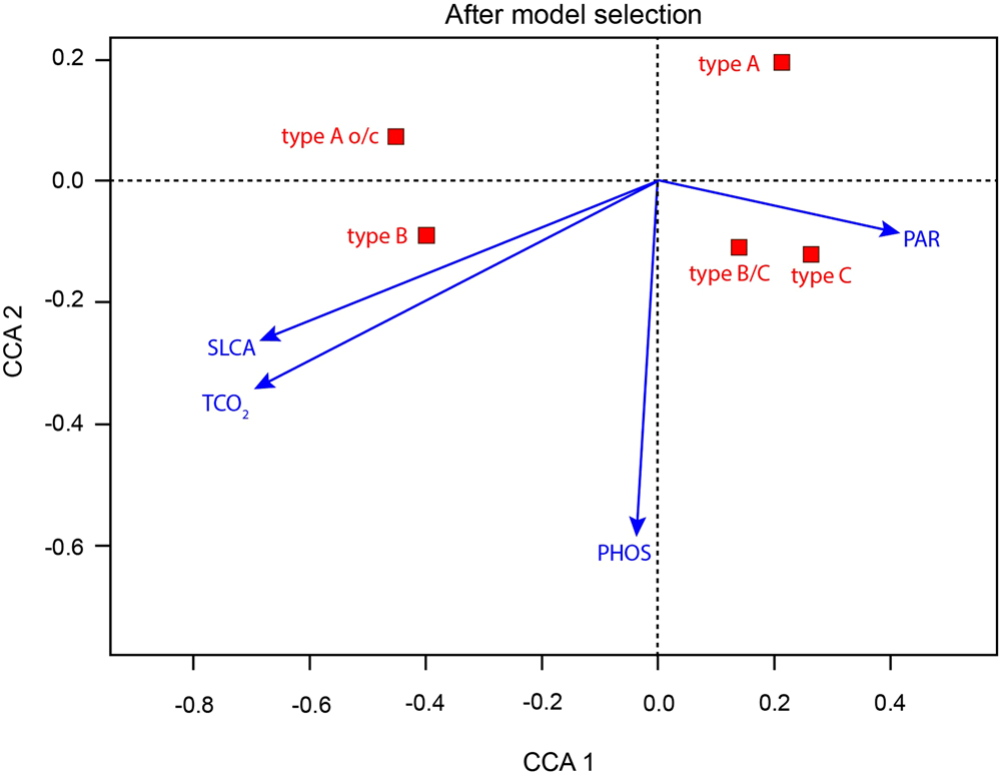
Canonical Correspondence Analysis (CCA) after model selection showing the correlation between the temporal changes in the relative abundance of *E. huxleyi* morphotypes collected by the autonomous water sampler and environmental factors (blue arrows) at SOTS. Arrows indicate the directions and relative importance (arrow lengths) of each environmental factor on each axis. SLCA – silicate; TCO_2_ – total dissolved inorganic carbon (TCO_2_); PHOS – phosphate; PAR – photosynthetically active radiation.

Canonical correlation analysis (CCA) results indicate that TCO_2_, phosphate, PAR and silicate alone account for 78% of the total inertia (i.e. the amount of variation) in the dataset (F[4,18] = 15.979, p = 0.001) (Fig. 7). The first axis explained 61% of the inertia and was mostly negatively related with TCO_2_ and positively with PAR. The second axis explained 13% of the variability and was mostly related with phosphate concentration (Fig. 7). Both type A o/c and B were related with high silicate concentration and TCO_2_, whereas type A was associated to low values of these two variables. Type C and type B/C were related with high values of PAR.

Further, the CCA evidenced three groups of morphotypes: (i) A o/c and B, (ii) C and B/C and (iii) A (Fig. 7), which have also distinctly different seasonal distributions (Fig. 5). Morphotypes A o/c and B exhibited their maximum relative abundance during the austral winter-spring transition 2011 and winter 2012 coinciding with minimum annual SST, Ωcalcite and pH, and maximum annual TCO_2_ and macronutrient concentrations (Fig. 5). In the case of the C and B/C group, both morphotypes reached maximum annual relative abundance between December and early February at the SOTS site, coinciding with the period of enhanced algal biomass accumulation in the surface layer and coccolithophore export in the sediment traps. In late summer (late February-March), Type A became the dominant morphotype (>56%) (Fig. 5a), coinciding with highest annual Ωcalcite and pH and lowest annual TCO_2_ and macronutrient concentrations. Lastly, on the commencement of winter in 2012, the relative contribution of Type A o/c in the *E. huxleyi* populations increased concomitantly with a reduction of Type A abundance, closing the annual cycle.

**Figure 8:**
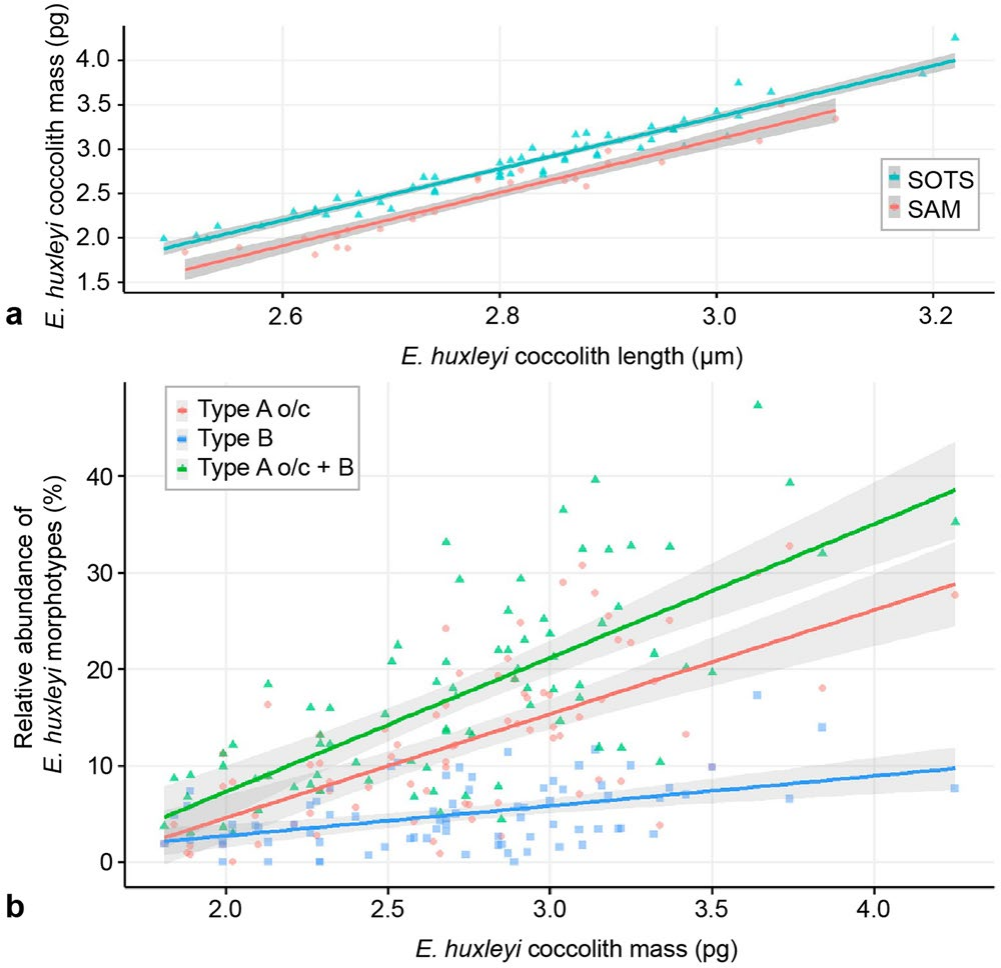
Correlation between coccolith mass and length at the SOTS and SAM sites (**a**) and between types A o/c and B morphotypes (%) and coccolith mass. Colour lines are fitted by regression and the grey shade denotes the 95% confidence interval for predicted mean values.

Coccolith mass was highly related with length (Mass = −5.691 + 2.928*Length, F[2,77] = 634.6, p < 0.001, R^2^ = 94%). The slope of the relationship did not differ between sites, but the intercept was significantly higher at the SOTS site than at the SAM site (Fig. 8a). Due to the high correlation between coccolith mass and length, only coccolith mass was compared with the morphotype composition of *E. huxleyi* populations.

The total coccolith mass was significantly related with the relative abundance of morphotype type A o/c and type B (Mass ~ 2.084 + 0.036 * type A o/c + 0.0384 * type B, F[2,77] = 39.53, p < 0.001), whereas the contribution of type B/C, type C and type A were not important, and thus excluded from the model (Fig. 8b). Both morphotypes explained 50% of the variability, with Type A o/c being more important than type B. In face of these results, a new model was built with the abundance of the two types summed, for comparability in future works (Mass ~ 2.087 + 0.037 * (Type A o/c + B), F[1,78] = 80.02, p < 0.0001, R^2^ = 50.6%), which gave very similar results, as expected.

## Discussion

Underpinning the tremendous capacity for adaptation of *Emiliania huxleyi* is its high genome variability, which drives phenotypic and physiological heterogeneity^48^. In particular, and relevant to our study, three different Southern Ocean *E. huxleyi* morphotypes, each of which characterized by different coccolith size and mass, have shown differing responses to changing *p*CO_2_ seawater chemistry conditions in laboratory culture experiments^28^. Therefore, documenting the diversity of *E. huxleyi* morphotypes in the SAZ and their seasonality in relation to changing environmental conditions is of critical importance to assess their response to projected environmental change in the Southern Ocean^5^.

The similar seasonality of types A o/c and B (Figs. 5 and 6) but distinctly different - and well-established - coccolith morphologies^49,50^ observed in the surface waters at the SOTS site indicate that they represent two different taxonomic varieties with relatively similar ecological niches. However, the case seems to be different for the group formed by morphotypes B/C and C. Because both morphotypes display similar seasonal distributions and the main discriminatory feature between them is coccolith size^49,50^, it is likely that types B/C and C in our samples represent different size-classes of the same ecotype. Our proposed classification of Subantarctic *E. huxleyi* morphotypes is, however, at slight variance with the approach of a recent comprehensive genetic study of 273 *E. huxleyi* clonal cultures from Australian and Southern Ocean waters^25^ where only two genetic varieties or ecotypes were discriminated: *E. huxleyi* var. *huxleyi* (i.e. Type A and A o/c) and *E. huxleyi* var. *aurorae* (i.e. Types C, B/C and B). These two ecotypes differ not only in coccolith morphology but also in photosynthetic pigment compositions and physiological response to environmental drivers^24,25^. There are two possible hypotheses to reconcile our field observations with Cook *et al*.’s genetical study: (i) morphotype A o/c and B represent overwintering phenotypes of *E. huxleyi* var. *huxleyi* and *E. huxleyi* var. *aurorae*, respectively as discussed in^51^ for A o/c, or (ii) the genetic differences between A and A o/c and between B/C and B may be too subtle to have been detected in^25^ study - focused on the *tuf*A chloroplast genes only.

In support of the first hypothesis are several culture studies that demonstrated that calcification in *E. huxleyi* is substantially less curtailed than photosynthesis under low irradiance levels, thereby leading to an increase in the inorganic carbon to organic carbon production ratio^30,52–54^. Thus, since both morphotype A o/c and B are known to be the morphotypes found in the Southern Ocean with highest coccolith mass^21,55^, their relative abundance increase during winter-spring could be caused by a physiologically driven change in coccolith weight of *E. huxleyi* var. *huxleyi* and *E. huxleyi* var. *aurorae*, respectively. Alternatively, evidence in favour of the second hypothesis is provided by^56^ who demonstrated that phenotypic variability between *E. huxleyi* strains is substantially greater than the phenotypic plasticity of single strains cultured under a wide range of environmental conditions. It follows that the amplitude of the seasonal cycle in average coccolith mass in the Subantarctic zone of ~2 pg, i.e. ~50% of the maximum coccolith mass (Fig. 4), is probably too large to be solely driven by a physiological change of a single *E. huxleyi* strain. Since no culture experiments, to the best of our knowledge, have been able to induce a change from Type A into Type A o/c (or vice versa) or Type B/C into B (or vice versa), we conclude that the different morphotypes found in the Subantarctic waters south of Australia and New Zealand must represent different ecotypes. The only exception seems to be types B/C and C, which have similar morphologies and seasonality, suggesting that they most likely correspond to the same ecotype.

The annual flux-weighted average mass of *E. huxleyi* coccoliths at the SOTS site is similar between the three sediment trap records (2.67–2.85 ± 1.5 pg) and about 0.4–0.6 pg heavier than those estimated for the SAM trap (2.28 ± 1.24 pg). Interestingly, the estimates for both the SOTS and SAM sites are higher than the annual average weight measured in the Antarctic Zone (AZ) south of Tasmania using the same birefringence-based approach (2.11 ± 0.96 pg, at 2000 m depth^57^;) (see also Fig. S4). The differences in *E. huxleyi* coccolith mass across these sites are mainly attributed to the different morphotype composition, a factor considered to account for most of the coccolith mass variability in the global ocean^26^. Indeed, the lowest coccolith mass observed in the AZ is consistent with the “monomorphotypic” composition of the *E. huxleyi* populations at this location that were solely composed of morphotype B/C, characterized by lighter coccoliths than those of type A, A o/c and B^19,21,55^. In turn, maximum coccolith calcite quotas observed at SOTS are attributed to the greater relative contributions of morphotypes A and A o/c (~ two- and three-fold, respectively) compared to that at the SAM site (Fig. S1).

The strong and significant correlation between the temporal variations in the relative abundance of Type A o/c and B and coccolith mass (Fig. 8) suggest that changes in the abundance of these morphotypes are the main drivers of the seasonal oscillation in coccolith morphometrics observed at the SOTS and SAM sites. These results are consistent, once again, with previous studies were the calcite quotas of both A o/c and B coccoliths were reported to be significantly greater than their Subantarctic counterparts (i.e. B/C)^21,55,57^. Moreover, our results are in agreement with recent findings in the Mediterranean Sea, where highest *E. huxleyi* coccolith mass was positively correlated with relative abundance of Type A o/c along an environmental gradient^58^. Taken together, all the above mentioned studies and our results underscore the crucial role of *E. huxleyi* morphotype composition in the control, both geographically and seasonally, on coccolith size and mass variability in the Southern Ocean.

A wide range of environmental parameters including salinity, irradiance, temperature and CO_2_ and macronutrient concentrations (mainly nitrate and phosphate) are known to influence the physiology (i.e. growth, photosynthetic and calcification rates) of isolated *E. huxleyi* strains^28,30,59,60^. However, the ecological effect of changing environmental factors on the makeup of coccolithophore communities in their natural habitat is largely unknown. *In situ* monitoring of environmental parameters and *E. huxleyi* populations at the SOTS site allow us to explore with unprecedent detail the mechanisms driving the seasonal alternation in morphotypes in the Subantarctic waters south of Australia.

Our CCA results suggest that changes in TCO_2_ and silicate concentration may represent the most important controls in seasonal variation of *E. huxleyi* morphotypes in the SAZ. These observations are consistent with previous monitoring studies in several settings of the Northern Hemisphere where peak annual abundances of morphotype A o/c cells were observed during winter, a period characterized by maximum annual *p*CO_2_^51,61,62^. Additionally, evidence from laboratory manipulation experiments with Southern Ocean *E. huxleyi* strains at constant temperature (14 °C) under nutrient replete conditions^28^ indicates that increasing *p*CO_2_ results in a pronounced decrease in the growth rates of both types B/C and A, while its effect on A o/c is almost negligible. Most notably, morphotype B/C was shown to nearly cease the production of coccoliths under high *p*CO_2_ conditions. Thus, a possible explanation for the winter increase in the relative abundance of A o/c could be the differing response of the *E. huxleyi* morphotypes to seasonal changes in *p*CO_2_. However, it should be noted that *p*CO_2_ range used in Müller *et al*.’s experiment (240 to 1750 µatm) was substantially larger than the amplitude of the seasonal cycle of *p*CO_2_ at the SOTS site (∼60 μatm). Therefore, it remains unclear if such a subtle change in *p*CO_2_ could induce a natural shift in the dominant *E. huxleyi* morphotypes. Altogether, these studies and our data suggest that seasonal changes in the carbonate system could be an important factor driving the seasonal alternation of *E. huxleyi* morphotypes in the global ocean, however the exact mechanisms linking both remain to be determined.

The strong positive correlation of type A o/c and B with silicate concentration variability (Figs. 6 and 7) is intriguing. In particular because although some coccolithophore species require Si for their calcification process^63^, this requirement is entirely absent in the family Noelaerhabdaceae, that includes the genus *Emiliania*. However, dissolved silicate is an important macronutrient involved in production and growth of other phytoplankton, notably diatoms^64^. Therefore, it may indirectly drive the seasonal succession of morphotypes. In the surface waters of the SAZ, silica becomes seasonally depleted during spring-summer due to diatom growth^41,65–67^. Since diatoms generally exhibit higher growth rates than coccolithophores when all resources are non-limiting, it is only during times of silica depletion when coccolithophores may take precedence^68^. Thus, silica concentration in the surface layer could be taken as an indicator of diatom abundance in the SAZ. It follows that the positive correlation of type A o/c and B with silica concentration may reflect a lower capacity of these morphotypes to compete with silicifying diatoms than their calcifying counterparts (i.e. types A and B/C).

Our CCA results (Fig. 7) suggest that the variability in PAR and phosphate concentration may also exert some degree of control over the seasonal distribution of morphotypes. *E. huxleyi* populations south of the Polar Front have repeatedly been documented to be solely or almost entirely composed of Type B/C (i.e. *E. huxleyi* var. *aurorae*) indicating that this morphotype is better adapted to the cold and low-light regime of the high-latitude Southern Ocean than its counterparts (i.e. *E. huxleyi* var. *huxleyi*)^17,24,30,57^. Therefore, the peak abundance of the “B/C-C group” during the summer - a period characterized by maximum annual solar irradiance - was somewhat unexpected. *E. huxleyi* has an exceptionally high affinity for phosphate but is generally considered a poor competitor for nitrate^69^. In fact, blooms of this species are generally associated with low phosphate but higher nitrate levels^70^. At the SOTS site, type A reaches maximum relative abundance coinciding with the period of minimum annual phosphate and nitrate levels during summer. Based on the CCA results (Fig. 7), it could be argued that, at times of low phosphate concentrations, type A may thrive better than its counterparts, particularly B/C that is well adapted to the cold and nutrient-rich waters south of the Polar Front^17^. However, the surface waters of the SOTS never experienced potentially limiting phosphate or nitrate levels (Fig. 5^71^), therefore this hypothesis seems unlikely. Indeed, this is probably the same reason why nitrate seems to be unimportant in the seasonal variation of morphotypes in our study despite being ranked the most important environmental driver controlling the growth, photosynthetic and calcification rates of a Southern Ocean strain of *E. huxleyi* Type A in a recent study by Feng, *et al*.^30^.

Lastly, it is important to note that although water temperature was excluded from the CCA due to its high correlation with other environmental parameters (Fig. 6), it has been proposed as a critical factor for the biogeographic distribution of *E. huxleyi* morphotypes (Buitenhuis *et al*., 2008; Bach *et al*., 2012) and therefore it could play a role in the seasonal succession of morphotypes. In this regard, unpublished results from Hallegraeff *et al*. demonstrate that Southern Ocean B/C strains can grow at 4, 10 and 17 °C, while types A and A o/c did not survive at 4 ^o^C. Therefore it is possible that the increase in the relative abundance of morphotype A at times of maximum sea surface temperature (Fig. 5) relates to the high energetic cost of producing heavy A type coccoliths which are preferentially generated at higher temperatures. In turn, lighter B/C type may have a broader competitive advantage under all other environmental scenarios in the low temperature Southern Ocean.

The similar seasonality of *E. huxleyi* morphotypes observed at two distinct settings within the SAZ during different years suggests that this seasonal pattern may be a ubiquitous feature of the circumpolar SAZ. The considerable seasonal covariation between environmental parameters makes it difficult to determine with certainty if changes in the carbonate system are the primary controllers of the observed alternation of morphotypes. Since laboratory manipulations suggest that that the annual amplitude carbonate system parameters in the SAZ is too low to induce a marked change in the physiological rates of *E. huxleyi* morphotypes^28^, we speculate that the cumulative effect of changes on several environmental drivers^30,72^ is the most probable cause of the seasonal variation in morphotypes. Moreover, our results add to the findings of previous studies in the Northern Hemisphere that reported a similar seasonal preference of heavily calcified *E. huxleyi* forms for high surface water *p*CO_2_ and low pH and Ωcalcite during winter^51,61,62^. All these studies together challenge the notion that the ongoing global ocean acidification will be detrimental for heavily-calcified coccolithophores^26^ while emphasizing the seasonal and spatial dominance of the weakly calcified B/C morphotype in the Southern Ocean.

## Supplementary information

Supplement information.

Supplement Table 1.

Supplement Table 2.

## Data Availability

Abundance, composition and morphometric data of *E. huxleyi* coccolith assemblages generated during the current study are listed in Supplement Table 1, while all the environmental data can be found in Supplement Table 2.
