# Supplementary material for: Full annual monitoring of Subantarctic Emiliania huxleyi populations reveals highly calcified morphotypes in high-CO2 winter conditions
Source: Sci Rep. 2020 Feb 13;10:2594. doi: 10.1038/s41598-020-59375-8 (PMC7018777; doi:10.1038/s41598-020-59375-8)
Supplement: Supplementary file 1 — Supplement information. [file 41598_2020_59375_MOESM1_ESM.pdf]

The following supplement accompanies the article:

Full annual monitoring of Subantarctic *Emiliania huxleyi* populations reveals highly calcified morphotypes in high-CO_2 winter conditions

A.S. Rigual-Hernández*, T.W. Trull, J.A. Flores, S. D. Nodder, R. Eriksen, D. M. Davies, G. M. Hallegraeff, F.J. Sierro, S. Patil, A. Cortina, A.M. Ballegeer, L. C. Northcote, F. Abrantes, M. M. Rufino.

*Corresponding author. Email: arigual@usal.es

1. Sample collections and sensor measurements

All sediment traps were McLane PARFLUX-type design with a honeycomb baffle at the top (0.5 m^2 surface area). The sediment traps had a microprocessor-controlled, rotating carousel for the collection of 21 samples with the exception of the 47°S 3800 m trap that was equipped with a 13-sample carousel. The 250 ml sediment trap sample bottles were filled prior to each deployment with a solution prepared with filtered seawater (0.7 µm GF/F) from the same location as the traps for the SOTS traps while filtered seawater collected from the depth of the trap was used in the case of the SAM traps. For both stations, the seawater used to fill the cups was treated with 5-6 g L^{-1} sodium chloride to increase solution density, 1 g L^{-1} sodium tetraborate as a pH buffer and poisoned with 3 g L^{-1} mercuric chloride.

The Remote Access Sampler (RAS 500; McLane Labs) collected 48 un-filtered 500mL samples into Tedlar® bags via a rotary multiport distribution valve with a common inlet. Prior to each sampling event, 100 mL of seawater was flushed through the inlet. After sampling, 4mL of biocide solution (0.5 mM mercuric chloride in deionized water) was flushed through the valve and out the inlet, to reduce biological growth[1,2]. Previous work in the North Pacific has shown that this device provides unbiased nutrient measurements [2]. On the Pulse mooring, the RAS sampler was placed inside a protective black plastic shroud and the inlet was routed to its exterior to a 1 mm^{-2} nylon mesh and a copper metal antifoul shield [1]. The narrowest section inside the RAS multi-port valve has a diameter of 1.39 mm. Samples were collected in pairs, one hour apart, every 15 days. The even numbered sample in each pair was preserved with 0.25 mL saturated mercuric chloride (to achieve ~ 80 µM final concentration), for analysis of nutrients, DIC and alkalinity. The odd numbered sample in each pair was preserved with 20 mL glutaraldehyde (to achieve ~1% w/w final concentration) for phytoplankton identification. Sample preservatives were loaded after priming with approximately 5 mL of boiled MilliQ water. Although the glutaraldehyde preserved samples contained well preserved coccoliths, because of concern regarding oxidation of the archived samples, the mercuric chloride preserved samples were used for this study. Nutrient concentrations (silicate, phosphate, and the Total oxidised nitrogen – TNOx –, i.e. sum of nitrate and nitrite) were measured on these same samples by flow injection analysis (Lachet 8000) against standard curves and international seawater standards in the CSIRO Hydrochemistry Facility, with precisions of a few percent. Small corrections (~1%) were applied to account for dilution by the de-ionized water used to pre-fill the RAS valve and tubing prior to deployment, which becomes entrained into the samples.

Sensor measurements from SOTS were used to provide context for the RAS sample collections as follows. PAR measurements were from a spherical sensor (MDS-MKVL2000655, Alec Inc.) mounted in-air on the Pulse-8 surface float. Surface mixed layer salinity was measured using a CTD (Seabird SBE16+ at 36 to 49 m depth) within the RAS package, but because this failed mid-deployment, data was used from a deeper sensor (Seabird SBE37 at 105 m depth - slightly below the mixed layer in summer, which showed excellent correlation and negligible offset with the RAS mounted sensor when both were operational). For the same reason, temperature was taken from a deeper sensor (SBE56 at 45 m depth - within the mixed layer). Chlorophyll-*a* was excluded from the analysis because the record ended in March 2012. Averages of all the available values for the 7 days prior collection of the sample by the RAS were estimated. Also values higher than three times the standard deviation were considered oultiers and therefore not used for the analysis.

In situ carbonate system measurements at the SOTS site were taken from Shadwick, et al. [3] for the period November 2011 to October 2013. As the coccolith sampling commenced in August 2011, the missing carbonate system data between August to November 2011 was completed with data from year 2012. The assumption that the seasonality of the carbonate system between adjacent years show little variability in the Subantarctic Zone (SAZ) is supported by previous work in the region [4,5]. Lowest TCO_2 concentrations were registered in mid-summer (~ 2060 μmol kg^{-1}; Figure 4) mainly driven by biological activity while and maximum concentrations (> 2100 μmol kg^{-1}) were registered in winter-spring transition when mixed layer *p*CO2 is in near-atmospheric equilibrium. Both pH and calcite saturation state displayed a nearly opposite pattern with annual maxima in summer (8.10 and 3.97) and minima in early spring (8.03 and 3.03; Fig. 4).

2. Regional representativeness of the SOTS and SAM sites

Comparison of remote sensing and hydrographic observations suggests that the High Nutrient Low Chlorophyll (HNLC) waters sampled by the SOTS site can be considered representative of a large portion (~90 to 145° E) of the Subantarctic Zone [1,6,7]. Similarly, the sediment trap on the SAM has an estimated particle source area ('statistical funnel') of ~120 km for a particle sinking speed of 100 m d^{-1} that potentially encompasses a wide area of the northern sector of the SAZ off eastern New Zealand [8,9]. More specific statements regarding the largescale representativeness of the morphometric *E. huxleyi* observations are not possible, although we note that sparse particulate inorganic carbonate abundance observations from ships and as derived from satellite reflectance show similar patterns over the SAZ south of Australia and New Zealand [10,11].

Importantly, oceanographic observations at the SOTS site and more generally in in the SAZ suggest that the relationships between *E. huxleyi* morphotypes and environmental conditions are likely to represent local adaptations rather than originate from advective transports to the region. Water parcels with different T-S properties are occasionally advected past the SOTS site (e.g. three periods with relatively warm and salty compositions were observed in the annual record presented by [12]. However, comparison to spatial variations obtained from satellite and ship observations suggests

that relatively short trajectories (~100 km) are sufficient to explain these variations, because the Subantarctic Zone waters around SOTS exhibit similar mesoscale variability [13]. Notably, the passage of these T-S anomalies does not exhibit any particular seasonality. Thus, while the passage of discrete water parcels may well contribute to the overall observed constellation of morphotypes, we do not have evidence that it influences their seasonality. More generally, SAZ surface water properties near SOTS do reflect the mixing of warm, salty, low DIC concentration waters supplied from the north (including via eddies released from the extension of the East Australian Current) with cold, fresh higher DIC concentration waters supplied from the south (via both Ekman and eddy tranports), [14,15]. These studies suggest that the balance of this mixing varies seasonally, with greater influence of the northern source in summer. Thus, the broadscale seasonality of advective inputs to the SAZ acts to reinforce the locally driven seasonality of warming and DIC availability, and thus advective inputs of E. huxleyi do not appear to be a viable alternate explanation for the seasonal variations in morphotypes.

3. Taxonomic descriptions of *Emiliania huxleyi* morphotypes

Morphotype identification of *E. huxleyi* coccoliths was based on taxonomic concepts of Young et al. (2003) and Hagino et al. (2005) with slight modifications concerning the size range of each morphotype adapted for the subantarctic populations analysed here. Coccoliths of *E. huxleyi* morphotype A are medium-sized (2.5-4 µm), with robust distal shield elements and clearly-visible central-area elements. Given the substantial variability in the degree of calcification of Type A coccoliths, the ratio between slit length (SL) and tube width (TW) [16] was used to classify them into two main categories (Fig. 2). Coccoliths with SL > TW were grouped as "regularly calcified" Type A, while Type A coccoliths with SL<TW or with a closed central area were classified under Type A over-calcified (o/c) (Fig. S1). Morphotype B coccoliths are large (3.5-5 µm), with numerous distal shield elements (≥35) and a central area open or sometimes covered with a thin plate. Type B/C coccoliths display a relatively similar morphology to types B and C but are intermediate in size (2.5-4 µm), usually with 25-33 distal shield elements with central area open or covered by a thin plate. In those few cases in which the length of the distal shield fell within the overlapping size range of B and B/C (i.e. between 3.5 and 4), the number of distal shield elements was used as distinguishing feature between these two morphotypes. Morphotype C coccoliths are small (≤2-3.5µm), often with irregular shape compared to other morphotypes, with a distal shield element number usually ranging between 18-25 and a central area open or covered by a thin plate. In those cases where the length of the distal shield fell within the overlapping size range of morphotypes B/C and C, the number of distal shield elements and shape of the coccolith (i.e. regular vs. irregular) were used as discriminative features.

4. Coccolith mass and size measurements

The birefringence-based method to provide coccolith mass and size estimates is based on the systematic relationship between the thickness of a given calcite particle and the interference colour produced under polarized light [17-19]. In this study, an apical rhabdolith of the genus *Acanthoica* collected by a sediment trap at the SOTS site was

used for calibration. The microscope light settings and camera parameters were kept constant throughout the imaging session. A calibration image of the same rhabdolith was taken at the beginning of each imaging session to account for possible bulb ageing. Additionally, the same *E. huxleyi* coccolith (termed "calibration coccolith") was imaged after every calibration in order to assess the consistency of the coccolith mass and length measurements between sessions. Images were then processed using C-Calcita software [17]. The output files for all calcite particles with a diameter ranging between 1 and 8 µm were visually examined and *E. huxleyi* coccoliths selected. The excellent coccolith preservation in the water and sediment trap samples allowed us to differentiate *E. huxleyi* coccoliths from species of genus *Gephyrocapsa* (i.e. the other members of Noëlaerhabdaceae family present in the SAZ with relatively similar coccolith shape and size range) on the basis of the presence or absence of a conjunct bridge. The standard deviation of the mass and length of the "calibration coccolith" was used as a measure of calibration error across sessions. The calibration error was ±5 and ±2 % for coccolith mass and length, respectively. Because our birefringence-based method uses grey scale images to estimate coccolith thickness, it can be applicable only to coccoliths thinner than 1.55 mm [20]. This does not represent a limitation in our analysis because the thickness of all *E. huxleyi* morphotypes are below this threshold.

The wide range of coccolith mass estimates proposed in the literature for *E. huxleyi* ranges between ca.1.4 and 7.0 pg [21,22]. The source of these variability is mainly due to two factors: differences in coccolith mass between morphotypes and methodological biases associated to each of the most commonly used techniques for coccolith mass estimation (i.e. morphometrics, regression and birefringence). Because morphotype B/C is the most abundant morphotype in our samples and is geographically restricted to the Southern Ocean [23,24], we limit the comparison of our results to studies conducted only in the Southern Ocean using the same methodology. It should be noted that this is a conservative approach, because a previous study in the AZ waters south of Tasmania [21] using our birefringence-based approach showed good agreement with previous estimates in the Southern Ocean obtained with morphometric and birefringence approaches [22,25].

5. Annual estimates

In order to facilitate comparisons with other settings, annual flux estimates were calculated. For the SOTS sediment trap records, the unobserved interval occurred in winter when fluxes were low and, therefore, annual estimates were obtained by using an average flux value of the first and last cups (both collected during the winter) to represent mean daily fluxes during the unobserved period. For the SAM sediment trap, the gaps over the collection interval were quasi-evenly distributed throughout the time series. In this case, the gaps in the record were filled by linear interpolation of the closest cups and the winter gap was filled using the same approach as in the SOTS time-series. Annual *E. huxleyi* coccolith mass and length estimates were estimated following the same approach. With regard to the annual contribution of *E. huxleyi* morphotypes, two different annual estimates were calculated: the average relative contribution of each *E. huxleyi* morphotype over the collection interval and the annualized flux-weighted relative

contribution of the morphotypes collected by the sediment traps. This information is illustrated in Figure S2.

6. Seasonal variations in coccolith mass and length

Monthly variability *E. huxleyi* coccolith mass and length measured at the sediment traps at the SOTS and SAM sites was evaluated using a Generalised Additive Model (GAM), to account for non-linear relationships, fitted with Gaussian distribution errors (WOOD). In particular, the relationships of coccolith mass and length with month were modelled using cyclic cubic regression splines, whose ends match, to take into account that after month 12 comes month 1. Stepwise model selection was carried out by AIC.

Month and site explained 74% of the variability in coccolith mass, and both variables were highly significant. Overall the seasonal variability in coccolith mass and length was similar for both sites, with maximum coccolith mass and length observed during September and lowest during February-March (Fig. S3).

7. Comparison analysis of *E. huxleyi* coccolith mass across depths and sites in the Southern Ocean

The variability in *E. huxleyi* coccolith mass across sites and depths of the SOTS, SAM (SAZ) and 61°S[26] (Antarctic Zone; AZ) sites was analysed using a linear model, assuming a Gaussian distribution of the dependent variable. Then model assumptions were verified visually. A full model was produced including all interactions (Mass = Site + Depth + Site:Depth). An automatic stepwise model selection procedure showed that there was no significant effect of depth, neither any of the interactions, thus the final model only included site (Fig. S4). The similar average annual coccolith weight observed at the three traps depths in the SOTS site together with the good preservation of the coccoliths suggested by SEM observations, in both SOTS and SAM sites, indicate that negligible coccolith dissolution occurs at meso- and bathypelagic depths in the study region or within the sediment trap cups.

Supplement figures.

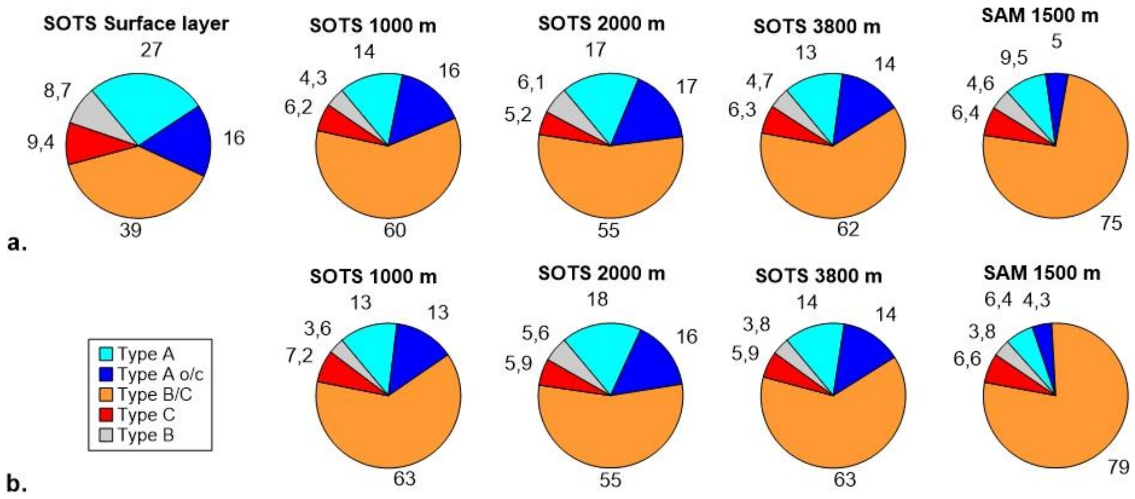

Figure S1: **a**. Average relative abundance of *Emiliania huxleyi* morphotypes over the collection intervals at the SOTS (surface layer, 1000 m, 2000 m and 3800 m depth) and SAM (1500 m) sites. **b**. Annualized flux-weighted relative contribution of *Emiliania huxleyi* morphotypes collected by the sediment traps at the SOTS and SAM sites.

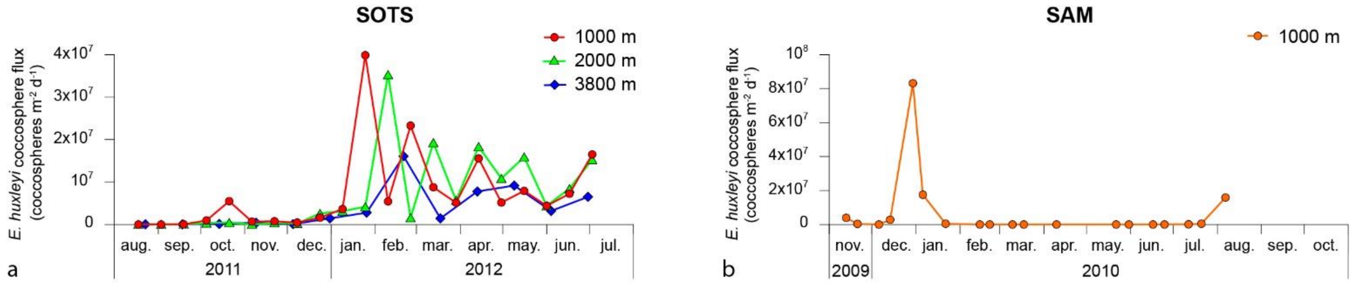

Figure S2: *Emiliania huxleyi* coccosphere fluxes collected by SOTS (a) and SAM (b) sediment traps.

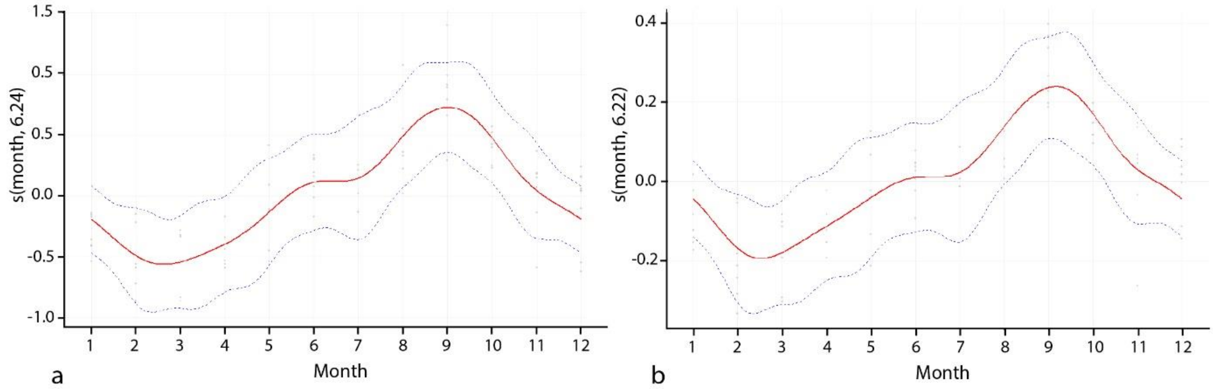

Figure S3: Monthly changes in coccolith mass (a) and length (b) using GAM using all the sediment trap data from the SOTS and SAM sites.

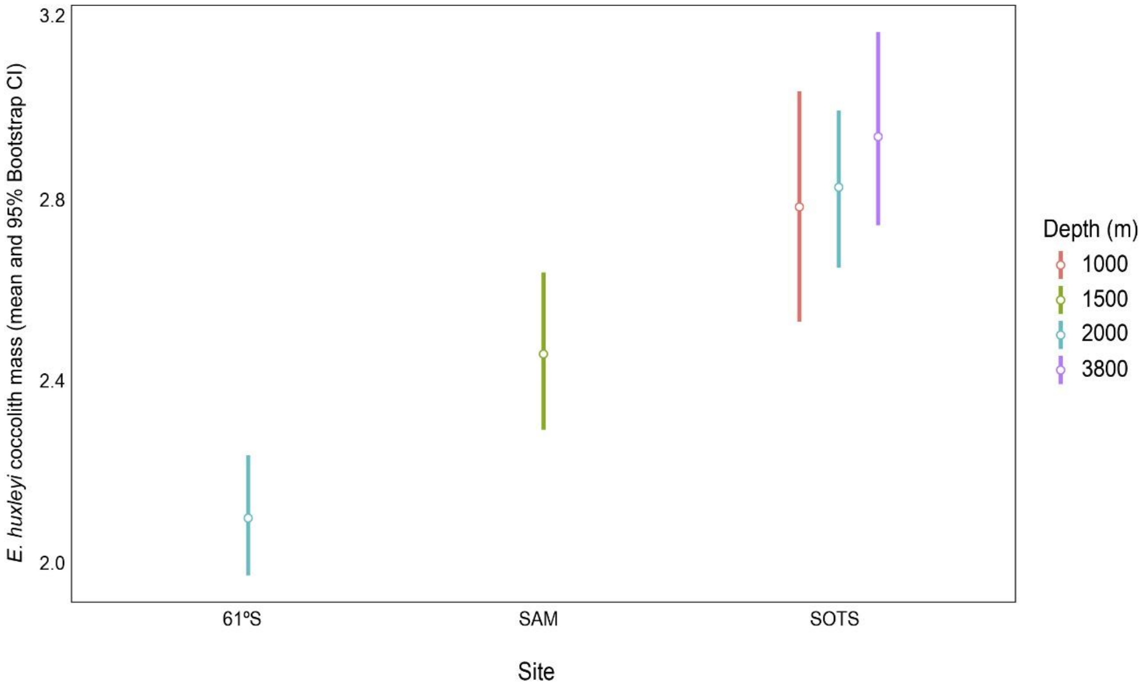

Figure S4: Changes in average *E. huxleyi* coccolith mass across sites and depths (mean and respective bootstrap 95% confidence interval). Coccolith mass changed significantly between the location of the sampling stations, with the different sites accounting for 30% of the variability in the masses ($F_{[2,97]}$=20.84, p-value < 0.001, R^2=30%). Site 61°S showed the smallest masses (2.10 ± 0.2 pg 95% CI), followed by site SAM (2.46 ± 0.18pg 95% CI), with SOTS site showing heaviest coccoliths (2.83 ± 0.12 pg 95% CI). Note that average coccolith mass values over the collection intervals (i.e. not annualized flux-weighted coccolith mass values) are presented in this figure.

Supplement Tables.

Table S1. **a**. Sampling dates and morphotype relative abundance of *E. huxleyi* coccolith assemblages collected in the surface layer at the SOTS site. **b**. Sampling intervals, fluxes and morphotype relative abundance and morphometric measurements of *E. huxleyi* coccolith assemblages intercepted by the sediment traps at the SOTS and SAM sites.

Table S2. Environmental parameters measured at the surface layer of the SOTS site from August 2011 to July 2012.

References

1 Eriksen, R. *et al.* Seasonal succession of phytoplankton community structure from autonomous sampling at the Australian Southern Ocean Time Series (SOTS) observatory. *Marine Ecology Progress Series* **589**, 13-31 (2018).

2 Honda, M. C. & Watanabe, S. Utility of an automatic water sampler to observe seasonal variability in nutrients and DIC in the Northwestern North Pacific. *Journal of Oceanography* **63**, 349-362, doi:10.1007/s10872-007-0034-5 (2007).

3 Shadwick, E. H. *et al.* Seasonality of biological and physical controls on surface ocean CO2 from hourly observations at the Southern Ocean Time Series site south of Australia. *Global Biogeochemical Cycles* **29**, 2014GB004906, doi:10.1002/2014GB004906 (2015).

4 Metzl, N., Tilbrook, B. & Poisson, A. The annual fCO2 cycle and the air–sea CO2 flux in the sub-Antarctic Ocean. *Tellus B* **51**, 849-861, doi:10.1034/j.1600-0889.1999.t01-3-00008.x (1999).

5 Takahashi, T. *et al.* Climatological mean and decadal change in surface ocean pCO2, and net sea–air CO2 flux over the global oceans. *Deep Sea Research Part II: Topical Studies in Oceanography* **56**, 554-577, doi:https://doi.org/10.1016/j.dsr2.2008.12.009 (2009).

6 Trull, T. W., Bray, S. G., Manganini, S. J., Honjo, S. & François, R. Moored sediment trap measurements of carbon export in the Subantarctic and Polar Frontal zones of the Southern Ocean, south of Australia. *Journal of Geophysical Research: Oceans* **106**, 31489-31509, doi:10.1029/2000JC000308 (2001).

7 Shadwick, E. H. *et al.* Seasonality of biological and physical controls on surface ocean CO2 from hourly observations at the Southern Ocean Time Series site south of Australia. *Global Biogeochemical Cycles* **29**, 223-238, doi:doi:10.1002/2014GB004906 (2015).

8 Nodder, S. D. *et al.* Temporal coupling between surface and deep ocean biogeochemical processes in contrasting subtropical and subantarctic water masses, southwest Pacific Ocean. *Journal of Geophysical Research: Oceans* **110**, n/a-n/a, doi:10.1029/2004JC002833 (2005).

9 Nodder, S. D., Chiswell, S. M. & Northcote, L. C. Annual cycles of deep-ocean biogeochemical export fluxes in subtropical and subantarctic waters, southwest Pacific Ocean. *Journal of Geophysical Research: Oceans* **121**, 2405-2424, doi:10.1002/2015JC011243 (2016).

10 Trull, T. W. *et al.* Distribution of planktonic biogenic carbonate organisms in the Southern Ocean south of Australia: a baseline for ocean acidification impact assessment. *Biogeosciences* **15**, 31-49 (2018).

11 Balch, W. M. *et al.* Factors regulating the Great Calcite Belt in the Southern Ocean and its biogeochemical significance. *Global Biogeochemical Cycles* **30**, 1124-1144, doi:10.1002/2016GB005414 (2016).

12 Weeding, B. & Trull, T. W. Hourly oxygen and total gas tension measurements at the Southern Ocean Time Series site reveal winter ventilation and spring net community production. *Journal of Geophysical Research: Oceans* **119**, 348-358, doi:10.1002/2013JC009302 (2014).

13 Shadwick, E. H., Tilbrook, B., Cassar, N., Trull, T. W. & Rintoul, S. R. Summertime physical and biological controls on O2 and CO2 in the Australian Sector of the Southern Ocean. *Journal of Marine Systems* **147**, 21-28, doi:https://doi.org/10.1016/j.jmarsys.2013.12.008 (2015).

14 C. Pardo, P. *et al.* Surface ocean carbon dioxide variability in South Pacific boundary currents and Subantarctic waters. *Scientific Reports* **9**, 7592, doi:10.1038/s41598-019-44109-2 (2019).

15 Herraiz-Borreguero, L. & Rintoul, S. R. Regional circulation and its impact on upper ocean variability south of Tasmania. *Deep Sea Research Part II: Topical Studies in Oceanography* **58**, 2071-2081, doi:http://dx.doi.org/10.1016/j.dsr2.2011.05.022 (2011).

16 D'Amario, B., Ziveri, P., Grelaud, M. & Oviedo, A. *Emiliania huxleyi* coccolith calcite mass modulation by morphological changes and ecology in the Mediterranean Sea. *PLOS ONE* **13**, e0201161, doi:10.1371/journal.pone.0201161 (2018).

17 Fuertes, M.-Á., Flores, J.-A. & Sierro, F. J. The use of circularly polarized light for biometry, identification and estimation of mass of coccoliths. *Marine Micropaleontology* **113**, 44-55, doi:http://dx.doi.org/10.1016/j.marmicro.2014.08.007 (2014).

18 Beaufort, L. Weight estimates of coccoliths using the optical properties (birefringence) of calcite. *Micropaleontology* **51**, 289-297, doi:10.2113/gsmicropal.51.4.289 (2005).

19 Beaufort, L., Barbarin, N. & Gally, Y. Optical measurements to determine the thickness of calcite crystals and the mass of thin carbonate particles such as coccoliths. *Nature Protocols* **9**, 633, doi:10.1038/nprot.2014.028

https://www.nature.com/articles/nprot.2014.028#supplementary-information (2014).

20 Bolton, C. T. *et al.* Decrease in coccolithophore calcification and CO2 since the middle Miocene. *Nat Commun* **7**, doi:10.1038/ncomms10284 (2016).

21 Rigual Hernández, A. S. *et al.* Coccolithophore populations and their contribution to carbonate export during an annual cycle in the Australian sector of the Antarctic zone. *Biogeosciences* **15**, 1843-1862, doi:10.5194/bg-15-1843-2018 (2018).

22 Poulton, A. J., Young, J. R., Bates, N. R. & Balch, W. M. Biometry of detached *Emiliania huxleyi* coccoliths along the Patagonian Shelf. *Marine Ecology Progress Series* **443**, 1-17 (2011).

23 Cubillos, J. *et al.* Calcification morphotypes of the coccolithophorid *Emiliania huxleyi* in the Southern Ocean: changes in 2001 to 2006 compared to historical data. *Marine Ecology Progress Series* **348**, 47-54 (2007).

24 Cook, S. S., Jones, R. C., Vaillancourt, R. E. & Hallegraeff, G. M. Genetic differentiation among Australian and Southern Ocean populations of the ubiquitous coccolithophore *Emiliania huxleyi* (Haptophyta). *Phycologia* **52**, 368-374, doi:10.2216/12-111.1 (2013).

25 Holligan, P. M., Charalampopoulou, A. & Hutson, R. Seasonal distributions of the coccolithophore, *Emiliania huxleyi*, and of particulate inorganic carbon in surface waters of the Scotia Sea. *Journal of Marine Systems* **82**, 195-205, doi:http://dx.doi.org/10.1016/j.jmarsys.2010.05.007 (2010).

26 Rigual Hernández, A. S. *et al.* Coccolithophore populations and their contribution to carbonate export during an annual cycle in the Australian sector of the Antarctic Zone. *Biogeosciences* **2017**, 1-40, doi:10.5194/bg-2017-523 (2018).
